# Onset-inhibition in the auditory brainstem: a potential mechanism for signal enhancement of speech-like sounds

**DOI:** 10.1186/1471-2202-14-S1-P148

**Published:** 2013-07-08

**Authors:** Martin J Spencer, David AX Nayagam, Janine Clarey, Hamish Meffin, Anthony N Burkitt, David B Grayden

**Affiliations:** 1 NeuroEngineering Laboratory, Dept of Electrical & Electronic Engineering, University of Melbourne VIC 3010, Australia; 2 National ICT Australia, c/- University of Melbourne VIC 3010, Australia; 3 Centre for Neural Engineering, University of Melbourne, VIC 3010, Australia; 4 Bionics Institute, East Melbourne VIC 3002, Australia; 5 Dept. of Pathology, University of Melbourne VIC 3010, Australia

## 

A prominent feature in the ascending auditory pathway is that of broadband onset inhibition, and there are many hypotheses regarding its functional role [1-4]. Here, we examine the possibility that it acts to suppress broadband onset splatter, the inharmonic noise that occurs at the beginning of sharp-onset sounds such as the glottal pulses in speech. We use experimental data from a circuit in the mammalian auditory brainstem formed by octopus cells in the posteroventral cochlear nucleus (PVCN), and their thick-axon projections to the ventral nucleus of the lateral lemniscus (VNLL) ending in calyx synapses. Octopus cells fire action potentials only in response to broadband auditory events, and they reliably stimulate cells located in the VNLL with a short latency. These cells, in turn, provide inhibitory connections to other VNLL cells (here referred to as the "VNLL cells"), which also receive excitatory input from cochlear nucleus (CN) cells [5]. We used a leaky integrate-and-fire (LIF) neuron model with experimentally constrained parameters to recreate the behavior of the circuit. The input to the model was provided by an existing auditory periphery model. A metric, the carrier clarity quotient (CCQ) was devised to quantify the signal strength of the carrier when modulated by a saw-tooth temporal envelope. This envelope is similar to that of glottal pulses in speech. It was observed that, at a certain relative delay between the excitatory and inhibitory input to the VNLL cells, the CCQ associate with a population of VNLL cells exceeded that of a comparable population of auditory nerve fibres (Figure 1).

**Figure 1:**
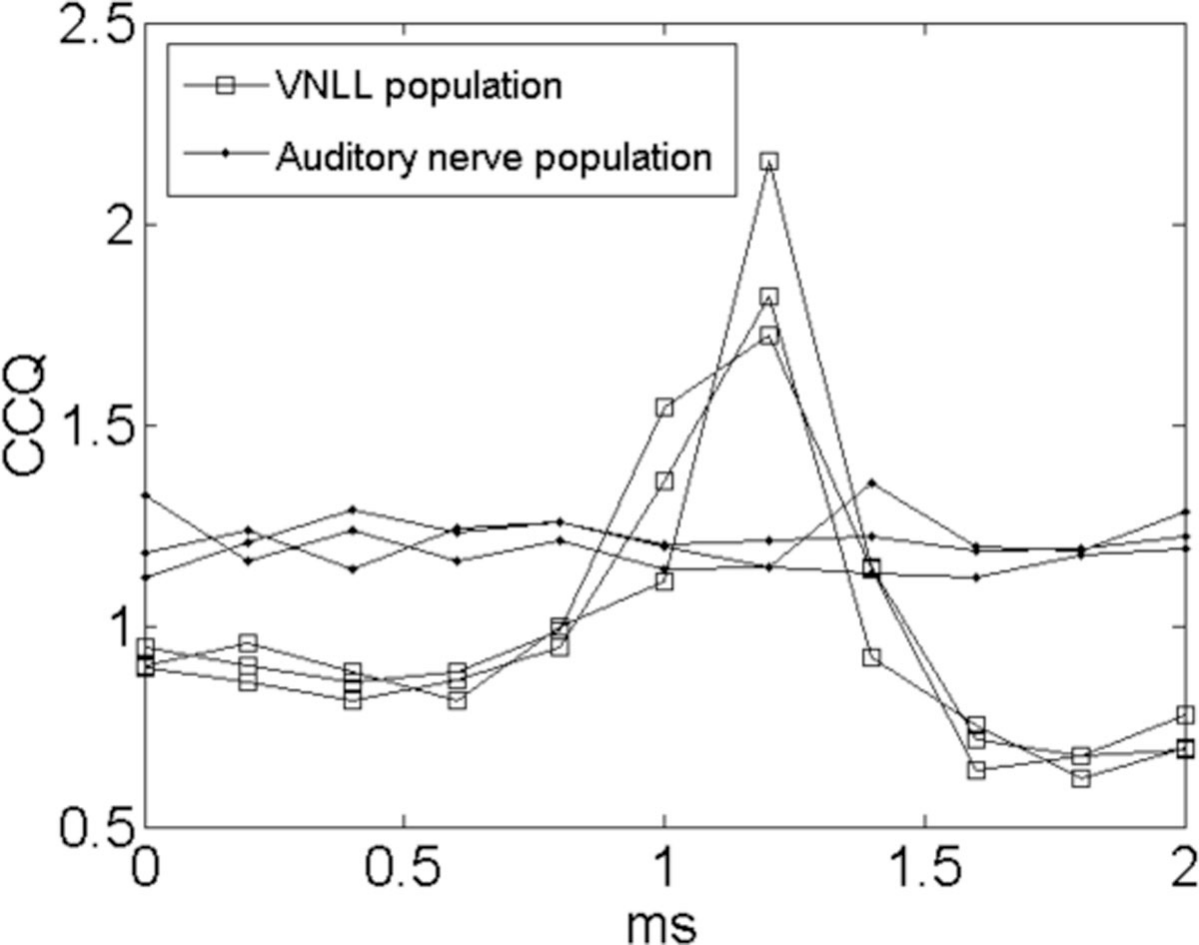
**The carrier clarity quotients for a modeled population of primary-like cells and Cell-C cells as a function of the relative delay between excitation and inhibition in the Cell-C population**. Three trials are plotted.

## Conclusion

Onset inhibition in the auditory brainstem can improve harmonic signal strength of speech sounds by suppressing the inharmonic onset noise associated with glottal pulses.
